# Supplementary material for: Drug repurposing for aging research using model organisms
Source: Aging Cell. 2017 Jun 16;16(5):1006–15. doi: 10.1111/acel.12626 (PMC5595691; doi:10.1111/acel.12626)
Supplement: Supplementary file 7 — Data S1 Zip‐Archive of all report cards. [file ACEL-16-1006-s007.zip › RC_HDI.pdf]

HDI

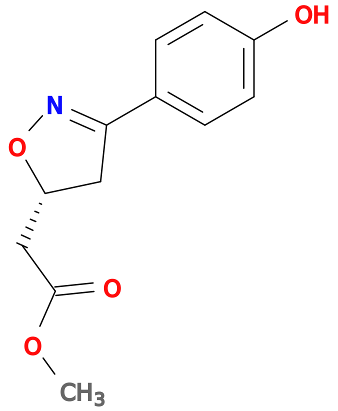

Database identifiers

ChEMBLCompound	CHEMBL211769
DrugBank	DB07888

Ranking

	Rank	Score
Drosophila	NA	NA
C. elegans	302/591	0.19

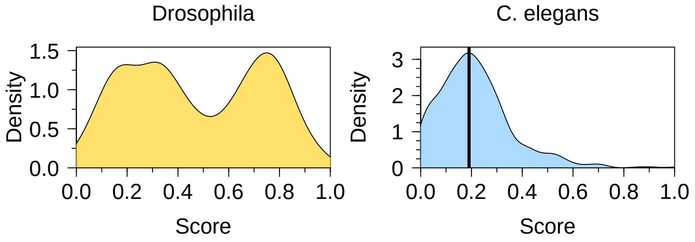

	Ageing implication	Domain conservation	Binding site conservation	Binding affinity	Bioavailability	Lipinski	Promiscuity	Purchasability	Drug approval	**Total**
Drosophila	NA	NA	NA	NA	NA	NA	NA	NA	NA	NA
C. elegans	0.81	0.855	0.885	0.736	0.257	0.0	-0.0	0.0	0.075	0.19

Names

- 2-(5R)-3-(4-keto-1-cyclohexa-2,5-dienylidene)isoxazolidin-5-ylacetic acid methyl ester
- 2-(5R)-3-(4-oxo-1-cyclohexa-2,5-dienylidene)-5-isoxazolidinylacetic acid methyl ester
- 3-(4-hydroxyphenyl)-4,5-dihydro-5-isoxazole-acetic acid methyl ester
- methyl 2-(5R)-3-(4-oxo-1-cyclohexa-2,5-dienylidene)-1,2-oxazolidin-5-ylacetate
- methyl 2-(5R)-3-(4-oxo-1-cyclohexa-2,5-dienylidene)-1,2-oxazolidin-5-ylethanoate

Roles

ChEBI entry None has no roles

Status

Approved drug (according to ChEMBL)	No
Classification (according to DrugBank)	experimental
Number of Rule of 5 violations	0
Binding affinity to original target in log units (RF-Score prediction)	6.02
Burns *C. elegans* bioavailability prediction	-1.79

Compound Target Characteristics

Macrophage migration inhibitory factor

Best gene implication in ageing for this target family came from gene P34884 annotated in UniProt release 2014_02. Annotation GO subterm of 7568 (aging) was Inferred from Mutant Phenotype

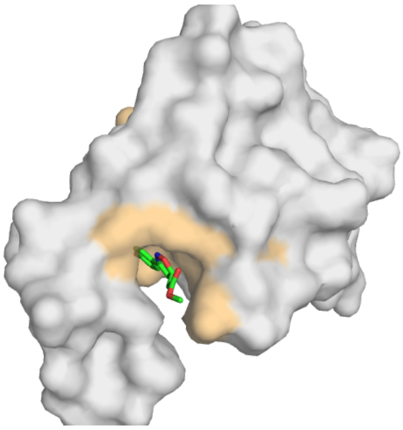

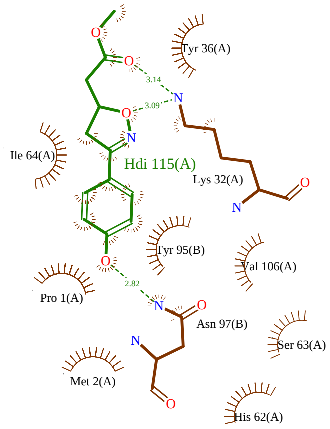

protein	amino acids contacts (binding site)									
PDB:1ljt:chainA:P14174	P	M	K	Y	H	S	I	Y	N	V
sp:P14174:MIF_HUMAN	P	M	K	Y	H	S	I	Y	N	V
tr:I4AY87:I4AY87_HUMAN	P	M	K	Y	H	S	I	Y	N	V
sp:P30904:MIF_RAT	P	M	K	Y	H	S	I	Y	N	V
tr:D3ZE63:D3ZE63_RAT	P	M	K	Y	H	S	I	Y	N	V
tr:D4A3P7:D4A3P7_RAT	P	M	K	Y	H	S	I	Y	N	V
sp:P34884:MIF_MOUSE	P	M	K	Y	H	S	I	Y	N	V
tr:Q545F0:Q545F0_MOUSE	P	M	K	Y	H	S	I	Y	N	V
tr:Q9U228:Q9U228_CAEEL	P	V	K	Y	K	S	I	Y	E	I
sp:Q18785:MIF2_CAEEL	P	M	K	R	K	S	I	V	T	V
tr:G3MU38:G3MU38_CAEEL	P	M	K	R	K	S	I	V	T	V

	whole protein		domain-based		contact-based	
protein	ident	simil	ident	simil	ident	simil
PDB:1ljt:chainA:P14174	1.0	1.0	1.0	1.0	1.0	1.0
sp:P14174:MIF_HUMAN	1.0	1.0	1.0	1.0	1.0	1.0
tr:I4AY87:I4AY87_HUMAN	1.0	1.0	1.0	1.0	1.0	1.0
sp:P30904:MIF_RAT	0.9	0.98	0.9	0.98	1.0	1.0
tr:D3ZE63:D3ZE63_RAT	0.86	0.96	0.86	0.96	1.0	1.0
tr:D4A3P7:D4A3P7_RAT	0.83	0.96	0.83	0.96	1.0	1.0
sp:P34884:MIF_MOUSE	0.9	0.97	0.89	0.97	1.0	1.0
tr:Q545F0:Q545F0_MOUSE	0.9	0.97	0.89	0.97	1.0	1.0
tr:Q9U228:Q9U228_CAEEL	0.33	0.78	0.33	0.78	0.6	0.88
sp:Q18785:MIF2_CAEEL	0.32	0.73	0.32	0.76	0.6	0.79
tr:G3MU38:G3MU38_CAEEL	0.27	0.62	0.32	0.76	0.6	0.79
